# Coagulation assays for assessing the bypassing of factor Xa direct oral anticoagulants using VMX-C001

**DOI:** 10.1016/j.rpth.2026.103375

**Published:** 2026-02-02

**Authors:** Magdolna Nagy, Tainá Gomes, René van Oerle, Tilman M. Hacking, Fabienne J.H. Magdelijns, Yvonne M.C. Henskens, Pieter Reitsma, Ged Short, Henri M.H. Spronk, Daniël Verhoef

**Affiliations:** 1 Coagulation Profile B.V., Maastricht, the Netherlands; 2 Department of Biochemistry, Cardiovascular Research Institute Maastricht, Maastricht University, Maastricht, the Netherlands; 3 VarmX B.V., Leiden, the Netherlands; 4 Division of General Medicine, Department of Internal Medicine, Section of Geriatric Medicine, Maastricht University Medical Center+, Maastricht, the Netherlands; 5 Cardiovascular Research Institute Maastricht, Maastricht University, Maastricht, the Netherlands; 6 Department of Clinical Chemistry, Maastricht University Medical Center+, Netherlands; 7 Division of Thrombosis and Hemostasis, Einthoven Laboratory for Vascular and Regenerative Medicine, Leiden University Medical Center, Leiden, Netherlands

**Keywords:** anticoagulation reversal, anticoagulation reversal agents, blood coagulation tests, direct factor Xa inhibitor, dilute prothrombin time, dilute Russell viper venom time, VMX-C001

## Abstract

**Background:**

Factor (F)Xa direct oral anticoagulants (DOACs) require rapid reversal in patients with serious bleeding or needing urgent surgery/interventions. VMX-C001 is a recombinant human FX variant under development as an FXa DOAC bypassing agent.

**Objectives:**

This study aimed to develop and evaluate the sensitivity of 2 modified commercially available coagulation assays—dilute prothrombin time (dPT) and dilute Russell viper venom time (dRVVT)—to assess coagulation with FXa DOACs with/without VMX-C001.

**Methods:**

Citrated platelet-poor plasma samples were obtained from healthy volunteers. Assay evaluation was performed using undiluted and 2.5× diluted plasma for dPT and 3× and 4× diluted plasma for dRVVT in the absence and presence of apixaban (100 or 250 ng/mL) and/or VMX-C001 (30 μg/mL). Additionally, dPT/dRVVT were measured in plasma spiked *in vitro* with apixaban, edoxaban, or rivaroxaban (0-1600 ng/mL) in the absence and presence of VMX-C001 (15-60 μg/mL), andexanet (50-200 μg/mL), or 4-factor prothrombin complex concentrates (0.25-0.50 IU/mL). *In vivo* assay evaluation used plasma samples from healthy subjects administered apixaban or rivaroxaban and from nursing home residents receiving FXa DOAC treatment.

**Results:**

Both modified dPT and dRVVT assays were highly sensitive to FXa DOACs, showing dose-dependent prolongation of clotting time, which was fully restored when VMX-C001 or andexanet (higher concentrations) was present in the plasma. Using both assays, clotting time from healthy subjects and nursing home residents receiving FXa DOACs correlated strongly with plasma drug concentrations.

**Conclusion:**

dPT and dRVVT assays can serve as surrogate end points in studies of VMX-C001 and can also be used to monitor FXa DOAC anticoagulation.

## Essentials

• Alternative and sensitive clotting assays for assessing bypassing of FXa DOACs by VMX-C001 are required.
• VMX-C001 is under clinical development as a bypassing agent for FXa DOACs.
• Modified dPT and dRVVT assays confirm bypass/reversal by VMX-C001 and andexanet, but not by 4F-PCC.
• Modified dPT and dRVVT assays are valid and sensitive for measuring FXa DOAC activity in patient plasma.

## 1 Introduction

Factor (F)Xa direct oral anticoagulants (DOACs) such as apixaban, rivaroxaban, and edoxaban are currently recommended as long-term treatment for the prevention of stroke in patients with nonvalvular atrial fibrillation and for the prevention and treatment of venous thromboembolism [[1], [2], [3], [4]]. FXa DOACs are administered at fixed doses, and routine laboratory monitoring of plasma levels is generally not required [5,6]. However, patients on FXa DOACs may require rapid reversal of their anticoagulation if they experience a serious bleeding event or require urgent surgery or invasive interventions.

Currently available rescue agents include andexanet alfa (andexanet) and prothrombin complex concentrates (PCCs). Andexanet is a specific reversal agent indicated for use in patients using apixaban or rivaroxaban who experience severe bleeding; it is currently not indicated for presurgery reversal. PCCs are indicated for reversal of vitamin K antagonist–associated major bleeding and are used off-label for treatment of FXa DOAC-related bleeding [7,8]. VMX-C001 is a modified recombinant variant of human FX under clinical development as a bypassing agent for FXa DOACs. In comparison with the native FX amino acid sequence, VMX-C001 has an insertion of 16 amino acids that replaces a stretch of 7 amino acids in the serine protease domain. VMX-C001 restores coagulation by replacing natural FX and is expected to be safe and effective in the case of bleeding or emergency surgery in patients treated with FXa DOACs [[9], [10], [11]]. As rapid establishment of normal haemostasis is desired in these situations, it would be useful to measure the level of anticoagulation using clotting assays to determine if reversal or bypassing agents are required [5].

Standard laboratory coagulation tests such as prothrombin time, activated partial thromboplastin time, and thrombin time lack sensitivity to assess the effects of FXa DOACs due to anticoagulant interference with these clot-based assays [5,12,13]. These coagulation assays have poor sensitivity for identifying clinically relevant FXa DOAC concentration thresholds (30-50 ng/mL), and results vary depending on the specific reagents used [12,14,15]. High-performance liquid chromatography-tandem mass spectrometry (LC-MS/MS) is the gold standard laboratory method for quantifying FXa DOAC plasma concentrations but is not feasible in routine clinical practice [5,16,17] and is not appropriate for assessing pharmacodynamic effects. Chromogenic anti-FXa assays are used to determine plasma levels of FXa DOACs, but they require calibration for each FXa DOAC and cannot be used to assess the effect of VMX-C001, which does not remove FXa DOACs from circulation [9,12,17,18]. In addition, in the acute setting, assessment of coagulation activity rather than FXa DOAC level is considered to be more useful.

Thus, there is a need for valid and sensitive laboratory assays to assess the effect of VMX-C001 on coagulation in FXa DOAC–treated patients presenting with severe bleeding or in those who require urgent surgery. Dilute prothrombin time (dPT) and dilute Russell viper venom time (dRVVT) are common tests for detecting lupus anticoagulant (LA) [19,20] and are available as commercial assay kits. Previous studies examining dPT as an assay for responses to FXa DOACs found that thromboplastin dilutions of 1:300 to 1:1500 showed linear dose-dependent prolongation of clotting times in plasma samples spiked with rivaroxaban (0-500 ng/mL) or apixaban (0-600 ng/mL) [13], with a good correlation between dPT values and plasma concentrations of the FXa DOACs [21]. Both Russell viper venom time and dRVVT are sensitive tests for the presence of FXa DOACs [22,23].

The primary objective of this study was to develop sensitive clotting assays for detection of coagulation responses following administration of VMX-C001 to patients receiving FXa DOACs. The secondary objective was to develop sensitive clotting assays for detection of coagulation responses to FXa DOACs. We modified the dRVVT and dPT clotting assays by using the high phospholipid reagent provided in the LA assay kits together with using different sample dilutions from those used in the standard LA assays. We evaluated the precision, accuracy, and sensitivity of these modified assays to the *in vitro* coagulation effects of FXa DOACs and restoration of coagulation by VMX-C001, andexanet, and 4-factor PCC (4F-PCC) in plasma from healthy volunteers and assay sensitivity in plasma from healthy older subjects and elderly nursing home residents receiving FXa DOACs. The aim was to demonstrate that tailored assays may be used to monitor anticoagulation in plasma and to establish the effectiveness of specific and nonspecific rescue agents of FXa DOACs.

## 2 Materials and Methods

### 2.1 Study design

The study consisted of 3 phases: (1) *in vitro* evaluation of the precision and accuracy (validity) of the modified dPT and dRVVT assays in normal human plasma alone or spiked *in vitro* with apixaban and/or VMX-C001; (2) determination of assay sensitivity in normal human plasma spiked *in vitro* with FXa DOACs and reversal of the anticoagulant effect with VMX-C001, andexanet, or 4F-PCC; and (3) assay evaluation using plasma samples collected from healthy older subjects administered FXa DOACs *in vivo* as part of the phase 1 study of VMX-C001 (NCT05152420) [11] and in plasma samples collected from nursing home residents receiving FXa DOAC treatment in an ongoing exploratory cohort study. All assays were performed at Coagulation Profile BV (Maastricht, the Netherlands).

The work described has been carried out in accordance with the Code of Ethics of the World Medical Association (Declaration of Helsinki). Ethics Committee approval was granted by Maastricht University Medical Centre for blood collection from healthy volunteers and from nursing home residents. Ethics Committee approval was present for the phase 1 study of VMX-C001 in healthy subjects. All subjects gave written informed consent.

### 2.2 Materials

For the *in vitro* experiments, apixaban and rivaroxaban were obtained from Alsachim (Shimadzu) and edoxaban was obtained from AdooQ Bioscience. Stock solutions (2 mg/mL) were prepared in dimethyl sulfoxide and stored at −80 °C. Dilutions of FXa DOACs were made using HEPES buffered saline (pH 7.4). For the phase 1 clinical study, apixaban 5 mg tablets Eliquis (Bristol Myers Squibb/Pfizer) and rivaroxaban 20 mg tablets Xarelto (Bayer Inc) were sourced from Alliance Healthcare.

VMX-C001 was provided by VarmX BV (batch C2103; 86.5 IU/mL, 10.3 mg/mL) and stored at −80 °C until diluted in HEPES buffered saline (pH 7.4) prior to use. Andexanet was purchased from AstraZeneca (batch 1695685; 10 mg/mL), dissolved in water for injection and stored in aliquots at −80 °C until use. The stock solution was used for experiments. 4F-PPC (CoFact) was purchased from Sanquin and dissolved in water for injection (batch 21A14G31A; 25 U/mL). The stock solution was used without further dilution.

The ACTICLOT dPT assay from BioMedica Diagnostics was used for the dPT assay and the HemosIL dRVVT Confirm assay from Instrumentation Laboratory (IL) Company was used for the dRVVT assay. Rotachrom/STA-Liquid Anti-Xa reagent and corresponding controls, and FXa DOAC–specific calibrators were purchased from Stago.

### 2.3 Plasma sample preparation

For the *in vitro* assay validity experiments, blood was obtained from 22 healthy volunteer donors and, for the *in vitro* assay sensitivity experiments, from 28 healthy volunteer donors (aged 18-65 years). This blood was used to prepare either normal pooled platelet-poor plasma (NPP) or individual platelet-poor plasma samples. The blood was collected by venipuncture into 3.2% (w/v) buffered sodium citrate and centrifuged at 2500 × *g* for 5 minutes at 18 °C. The plasma was then removed and spun for 10 minutes at 10,000 × *g* to obtain platelet-poor plasma, which was either pooled or stored individually at −80 °C until analyzed. Frozen plasma was thawed for at least 10 minutes at 37 °C and used within 4 hours. NPP was used for the reference or quality control (QC) plasma samples.

Blood samples were collected from healthy older subjects (aged 50-79 years) participating in the phase 1 randomized, placebo-controlled study of VMX-C001, who had received a 3.5-day course of apixaban (5 mg twice daily; *n* = 8) or rivaroxaban (20 mg once daily; *n* = 5) and, 5 minutes after their last dose of FXa DOAC, placebo, and from nursing home residents receiving treatment with rivaroxaban (*n* = 17), apixaban (*n* = 30), or edoxaban (*n* = 8).

For the dPT/dRVVT assays, citrated plasma samples were obtained from each subject immediately before the final dose of FXa DOAC (at trough), at the end of placebo infusion, and at various times up to 144 hours after the start of placebo infusion. Venous blood was collected into 4.3 mL citrate (3.2% w/v) S-Monovette 9NC collection tubes and centrifuged at 2500 × *g* for 10 minutes at 18 °C. Plasma was then transferred into 15-mL Greiner tubes and centrifuged at 2500 × *g* for 20 minutes at 18 °C. The resulting plasma was transferred into 2-mL Sarstedt PP storage tubes and stored frozen at −70 °C or lower until used for the dPT/dRVVT assays.

### 2.4 dPT assay

In the ACTICLOT dPT assay, the extrinsic coagulation pathway is activated with tissue factor (TF) in the presence of calcium ions. We modified the dPT assay to assess clotting time in the presence of FXa DOACs with and without VMX-C001 present in undiluted and 2.5× diluted human plasma. All analyses were performed using 2 Siemens BCSxp automatic analyzer systems, which were compared. For the undiluted test plasma, 80-μL plasma was supplemented with 40-μL prewarmed ACTICLOT LA phospholipids reagent and incubated for 2 minutes at 37 °C. Then, 40-μL prewarmed ACTICLOT dPT Activator reagent (containing a mixture of recombinant human TF, calcium, and phospholipids) was added and the clotting time recorded in seconds. For the 2.5× diluted test plasma, 30-μL plasma was diluted with 50-μL HEPES buffer, and then, 40 μL each of the phospholipid and activator reagents were added as deacribed earlier, and the clotting time was recorded.

### 2.5 dRVVT assay

The HemosIL dRVVT LA assay kit provides a Confirm reagent that contains Russell viper venom, phospholipids, calcium, polybrene (neutralizes heparins), buffers, stabilizers, dyes, and preservatives. We used the Confirm reagent to quantitatively assess the presence of FXa DOACs and the effects of the reversal agent VMX-C001 in human plasma (3× or 4× diluted). All assay reagents were prepared according to the manufacturer’s instructions, and all analyses were performed using 2 Siemens BCSxp automatic analyzer systems.

For the 3× diluted test, 25-μL plasma was supplemented with 50-μL factor diluent and 75-μL Confirm reagent, and the clotting time was recorded. For the 4× diluted test, 20-μL plasma had 60-μL factor diluent and 80-μL Confirm reagent, and the clotting time was recorded.

### 2.6 *In vitro* assay validity evaluation

The validity (precision and accuracy) of the dPT and dRVVT assays was assessed in QC samples, which consisted of NPP samples in the absence or presence of apixaban (100 or 250 ng/mL) and/or VMX-C001 (30 μg/mL; approximately 0.3 IU/mL). The clotting time for each QC sample was measured in 6 replicates across 10 independent runs on 5 different days, and normal ranges were determined. The clotting times for NPP samples alone and in the presence of apixaban (100 or 250 ng/mL) were analyzed using 2 different Siemens BCSxp systems and compared.

Interindividual variation in clotting times was assessed in individual plasma samples from 22 healthy individuals in the presence and absence of apixaban (200 ng/mL). The maximum clotting time was set at 250 seconds for dRVVT and 300 seconds for dPT, and outliers with values above these maximums were removed. Individual plasma sample data were accepted if the clotting times for the unspiked QC plasma samples were within predefined acceptance ranges.

### 2.7 *In vitro* sensitivity experiments

NPP samples from healthy volunteers were spiked *in vitro* with apixaban, edoxaban, or rivaroxaban at final concentrations of 0 to 1600 ng/mL in the absence and presence of VMX-C001 (15, 30 or 60 μg/mL), andexanet (50, 100, or 200 μg/mL) or 4F-PCC (0.25 or 0.5 IU/mL), and dPT and dRVVT were measured using the assays and dilutions described above. The dPT and dRVVT assays were considered suitable if the unspiked QC NPP samples had clotting times within the predefined acceptance ranges.

### 2.8 dRVTT and dPT measurements in healthy older subjects and nursing home residents receiving FXa DOACs

dPT and dRVVT were measured using the assays described earlier in citrated plasma samples taken from healthy older subjects who had received apixaban or rivaroxaban. The dPT assay was performed in 2.5× diluted plasma and the dRVVT assay in 4× diluted plasma on BCSxp automatic analyzer systems. FXa DOAC levels were also measured.

In plasma samples from nursing home residents receiving FXa DOAC treatment the dPT assay was performed in 2.5× diluted plasma and the dRVVT assay in 4× diluted plasma on a Siemens CS-2500 coagulation analyzer, in parallel with measurements of FXa DOAC levels.

### 2.9 Determination of plasma FXa DOAC levels

Blood samples were collected from healthy older subjects for the measurement of FXa DOAC plasma concentrations using LC-MS/MS assay. Citrated blood samples were collected before FXa DOAC administration on day 4 and at 30 minutes and 1, 2, 4, 8, 12, and 24 hours after administration, centrifuged for 15 minutes at 1500 × *g* at 18 °C, and the resultant plasma was stored frozen at −70 °C or lower. The FXa DOAC LC-MS/MS assay was performed by Ardena Bioanalysis BV. FXa DOACs were extracted from the citrated plasma (100 μL) by off-line solid phase extraction and analyzed using an API 4000 LC-MS/MS system. FXa DOACs were quantified using isotope labeled internal standards obtained from Alsachim.

For the plasma collected from nursing home residents on FXa DOACs, plasma concentrations (anti-Xa level) of rivaroxaban, apixaban, and edoxaban were measured with the Rotachrom/STA-Liquid Anti-Xa reagent on a Siemens CS-2500 coagulation analyzer. During measurements, controls and FXa DOAC–specific calibrators were applied to calculate the FXa DOAC concentrations.

### 2.10 Statistical analysis

All statistical calculations and graphs were constructed using GraphPad Prism (10.2.3; GraphPad Software). Data are presented as descriptive statistics, with box plots drawn where appropriate. Clotting times between samples with and without an FXa DOAC present were compared using the Wilcoxon text. The nonparametric Friedman test was used for multiple comparisons of paired groups. A 2-way analysis of variance (anova) was used to compare the difference between mean clotting times for samples with and without VMX-C001. *P* values of < .05 were considered statistically significant.

## 3 RESULTS

### 3.1 *In vitro* assay validity evaluation

The precision and accuracy of the modified dPT and dRVVT clotting assays in NPP QC samples *in vitro* are summarized in Supplementary Table 1. The dPT clotting time had within-run coefficients of variation (CV) of <2% in undiluted plasma and <4% in 2.5× diluted plasma, irrespective of the presence or absence of apixaban (100 or 250 ng/mL) and/or VMX-C001 (30 μg/mL). The overall accuracy and precision were <7% in both undiluted and 2.5× diluted plasma. The dRVVT clotting time for the QC plasma samples had within-run CVs of <2% for both the 3× diluted plasma and <4% for 4× diluted plasma, irrespective of the presence or absence of apixaban (100 or 250 ng/mL) and/or VMX-C001 (30 μg/mL). The overall accuracy and precision was <5% in both the 3× and 4× diluted plasma. The 2 BCSxp analyzer systems were internally validated as comparable, with between-run CV of <5%.

**Figure 1:**
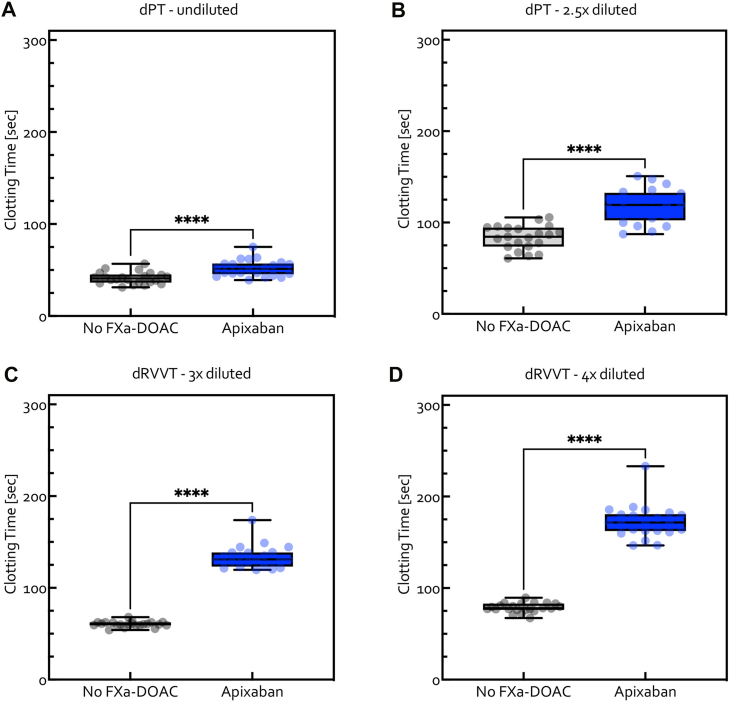
Interindividual variation and population averages for dPT clotting times measured in (A) undiluted and (B) 2.5× diluted individual plasma samples from healthy volunteers and for dRVVT clotting times in (C) 3× diluted and (D) 4× diluted individual plasma samples from healthy volunteers in samples without FXa DOAC (gray box plots) and spiked *in vitro* with apixaban 200 ng/mL (blue box plots). One subject with a clotting time above the limit of 300 seconds was removed from (B) as an outlier. ∗∗∗∗*P* < .0001 Apixaban vs no FXa DOAC (Wilcoxon test). DOAC, direct oral anticoagulant; dPT, dilute prothrombin time; dRVVT, dilute Russell viper venom time; FXa, factor Xa.

The dPT clotting times in the individual plasma samples in the absence and presence of apixaban (200 ng/mL) are shown in Figure 1A, B. For undiluted plasma, the mean dPT was 40.4 seconds (SD, 6.1 seconds; 95% CI, 38.0-42.8 seconds) in the absence of an FXa DOAC and 52.0 seconds (SD, 8.9 seconds; 95% CI, 48.0-55.9 seconds) in the presence of apixaban. For 2.5× diluted plasma, the mean dPT was 83.4 seconds (SD, 16.0 seconds; 95% CI, 77.2-89.6 seconds) and 126.6 seconds (SD, 42.7 seconds; 95% CI, 107.7-145.5 seconds) in the absence and presence of apixaban, respectively.

The dRVVT clotting times in plasma samples with and without *in vitro* spiking with apixaban (200 ng/mL) are shown in Figure 1C, D. For 3× diluted plasma, the mean dRVVT was 60.0 seconds (SD, 3.0 seconds; 95% CI, 58.9-61.2 seconds) in the absence of apixaban and 133.6 seconds (SD, seconds 12.2; 95% CI, 128.1-139.0 seconds) in the presence of apixaban. For 4× diluted plasma, the mean dRVVT was 78.0 seconds (SD, 5.2 seconds; 95% CI, 76.0-80.0 seconds) and 172.5 seconds (SD, 18.2 seconds; 95% CI, 164.4-180.5 seconds) in the absence and presence of apixaban, respectively. These *in vitro* experiments showed the validity of the modified dPT and dRVVT clotting assays for assessing clotting in the presence or absence of apixaban in human plasma.

### 3.2 *In vitro* sensitivity experiments

Dose-dependent prolongation of clotting times was observed with increasing doses of apixaban, rivaroxaban, and edoxaban in the modified dPT and dRVVT assays, irrespective of sample dilution (dPT: undiluted and 2.5×; dRVVT: 3× and 4×) (Supplementary Figure 1).

**Figure 2:**
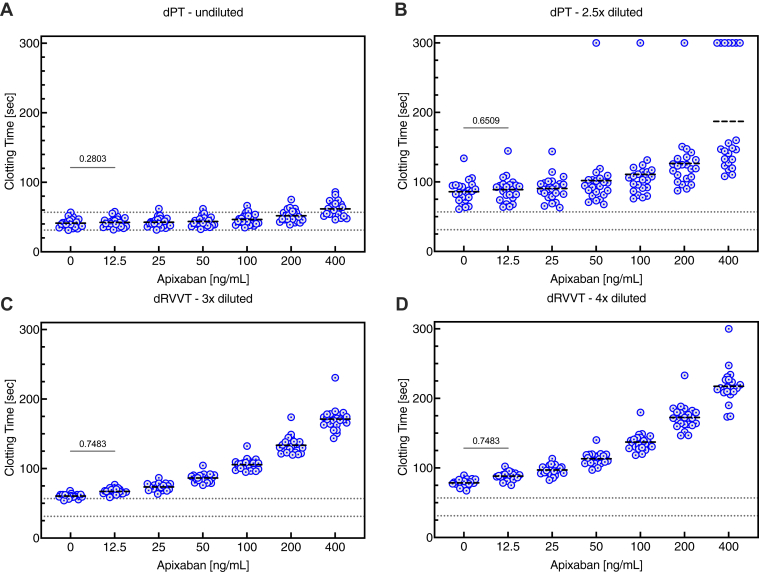
Effect of apixaban on dPT and dRVVT assays. Clotting times were obtained in (A) undiluted and (B) 2.5× diluted plasma for dPT and in (C) 3× and (D) 4× diluted plasma for dRVVT in individual plasma samples from 22 healthy volunteers spiked *in vitro* with apixaban (0-400 ng/mL). The dotted lines represent the minimum and maximum values of clotting times in the absence of apixaban (control). In all panels, the clotting times for each concentration of apixaban were compared with those for the samples without apixaban using the paired Friedman test for multiple comparisons. All comparisons were significant (*P* < .05, not indicated in the figure) except for those indicated in the figure. dPT, dilute prothrombin time; dRVVT, dilute Russell viper venom time.

Clotting times of plasma samples from individual healthy volunteers spiked *in vitro* with various concentrations of apixaban (0-400 ng/mL), obtained using dPT and dRVVT, showed a concentration-dependent prolongation of dPT with apixaban that was significantly different from the control range at apixaban concentrations of 25 ng/mL and higher (Figure 2A, B). In contrast, the concentration-dependent prolongation of dRVVT with apixaban was curvilinear, showing a higher sensitivity at low drug levels (25-50 ng/mL) (Figure 2C, D). It was significantly above the normal reference range of the assay (in the absence of apixaban), especially in the 4× diluted plasma. Overall, these data suggest that the dRVVT assay could be used to detect apixaban even at trough levels. Rivaroxaban and edoxaban gave similar results to those for apixaban (Supplementary Figure 2).

**Figure 3:**
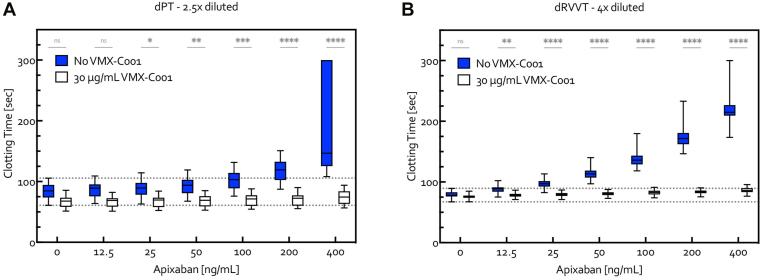
Evaluation of the (A) dPT and (B) dRVVT assays measured in 2.5× and 4× diluted plasma, respectively, in individual plasma samples from 22 healthy volunteers spiked *in vitro* with apixaban (0-400 ng/mL) without (control) and with VMX-C001 (30 μg/mL). The clotting time upper limit was 300 seconds. The dotted lines represent the minimum and maximum values for the control samples with 0-ng/mL apixaban. Comparisons between samples with and without VMX-C001 at each concentration of apixaban were made using a 2-way anova for multiple comparisons: ∗*P* < .05; ∗∗*P* < .01; ∗∗∗*P* < .001; ∗∗∗∗*P* < .0001. dPT, dilute prothrombin time; dRVVT, dilute Russell viper venom time; ns, not significant.

VMX-C001 was able to completely bypass the anticoagulant effect of apixaban, as demonstrated by reversal of the concentration-dependent prolongation of dPT and dRVVT with apixaban when VMX-C001 (30 μg/mL) was added to the plasma, resulting in normal clotting times (Figure 3). Similar results were achieved with rivaroxaban and edoxaban (Supplementary Figure 3).

**Figure 4:**
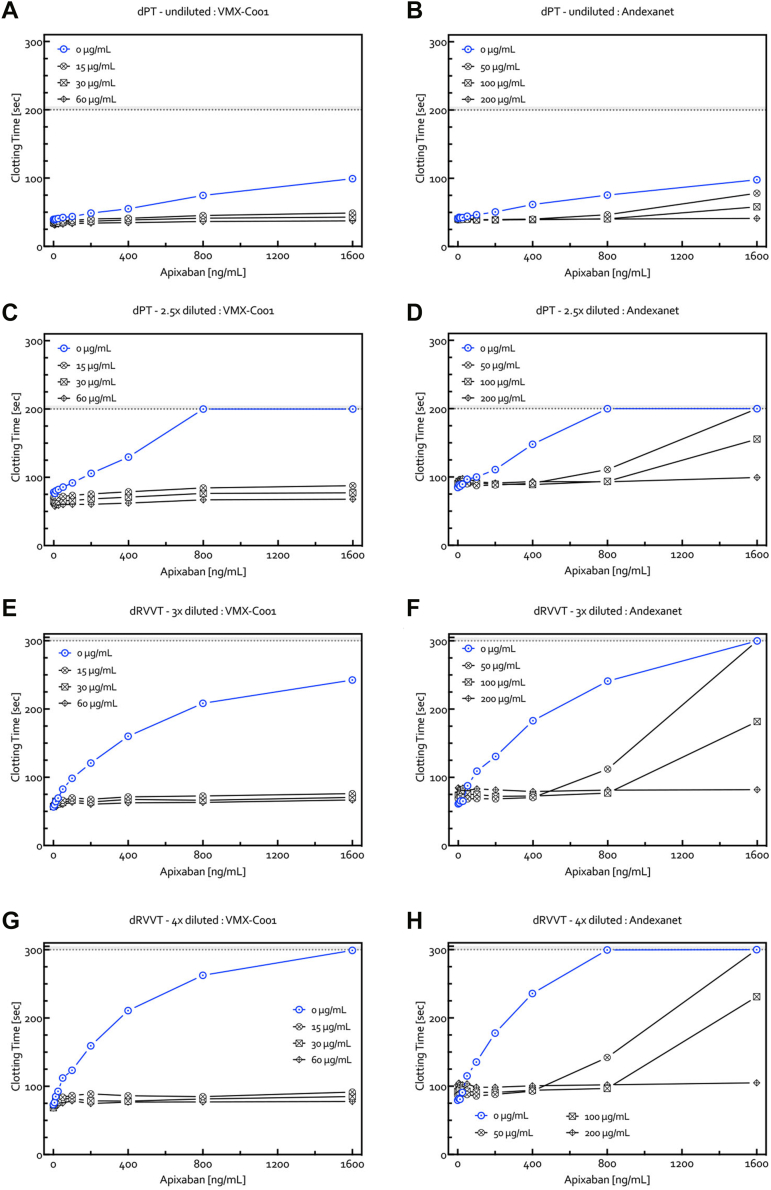
Dose-dependent reversal by VMX-C001 (15, 30, and 60 μg/mL) (A, C, E, and G) and andexanet (50, 100, and 200 μg/mL) (B, D, F, and H) of the dose-dependent prolongation of the dPT (A–D) and dRVVT (E–H) clotting times measured in NPP spiked *in vitro* with apixaban (0-1600 ng/mL). Dotted lines represent maximal readout times for both assays. dPT, dilute prothrombin time; dRVVT, dilute Russell viper venom time; NPP, normal pooled platelet-poor plasma.

**Figure 5:**
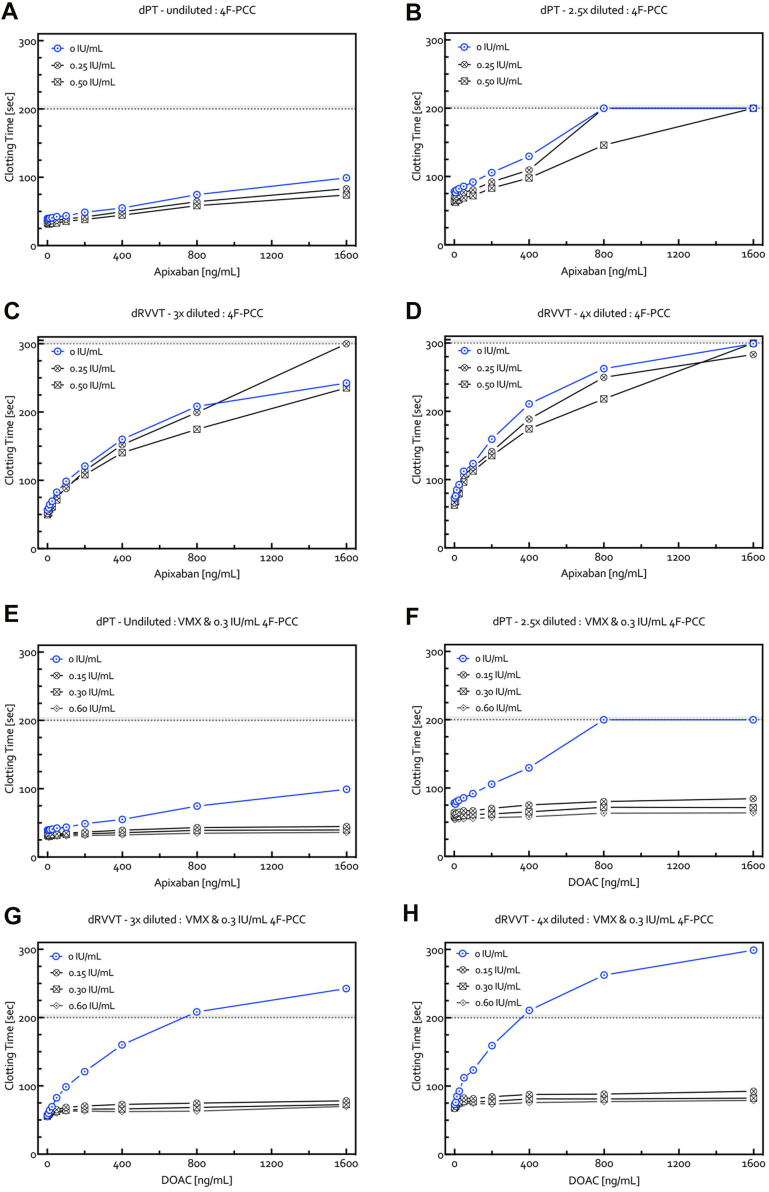
Effect of 4F-PCC on apixaban-induced prolongation of dPT (A, B) and dRVVT (C, D) clotting times in normal pooled plasma samples spiked *in vitro* with 4F-PCC (0.25 and 0.5 IU/mL) and increasing concentrations of apixaban (0-1600 ng/mL). (E‒H) The dose-dependent reversal by VMX-C001 of the prolonged clotting times in the presence of 4F-PCC (0.30 IU/mL). 4F-PCC, 4-factor prothrombin complex concentrates; dPT, dilute prothrombin time; dRVVT, dilute Russell viper venom time.

The reversal effects of VMX-C001 (15-60 μg/mL) and andexanet (50-200 μg/mL) were examined using the modified dPT and dRVVT assays with concentrations of apixaban up to 1600 ng/mL (Figure 4). While full reversal of dPT and dRVVT prolongation was seen with all doses of VMX-C001 for all concentrations of apixaban, this was only observed for the highest dose of andexanet (200 μg/mL). Lower doses of andexanet (50 or 100 μg/mL) resulted in full reversal of dPT/dRVVT prolongation only at the lower concentrations of apixaban (400-800 ng/mL). Similar results were obtained for VMX-C001 and andexanet with edoxaban (Supplementary Figure 4) and rivaroxaban (Supplementary Figure 5). In contrast, 4F-PCC was unable to normalize the apixaban dose-dependent prolongation of dPT and dRVVT clotting times as only minor improvement in clotting was seen with either 0.25 or 0.50 IU/mL 4F-PCC (Figure 5A–D). The results were similar with rivaroxaban and edoxaban (data not shown). When VMX-C001 was also present in the plasma sample with 4F-PCC (0.3 IU/mL), there was a full reversal of the prolonged clotting times with apixaban (Figure 5E–H). Similar results were obtained with rivaroxaban and edoxaban (data not shown).

Taken together, these *in vitro* spiking experiments showed that the 2.5× dilution for dPT and 4× dilution for dRVVT were the most sensitive in detecting FXa DOAC anticoagulation. These dilutions were therefore used for assaying plasma samples from healthy subjects receiving apixaban or rivaroxaban, and nursing home residents were treated with FXa DOACs *in vivo*.

### 3.3 Evaluation of dPT and dRVVT in healthy older volunteers and nursing home residents receiving FXa DOACs

**Figure 6:**
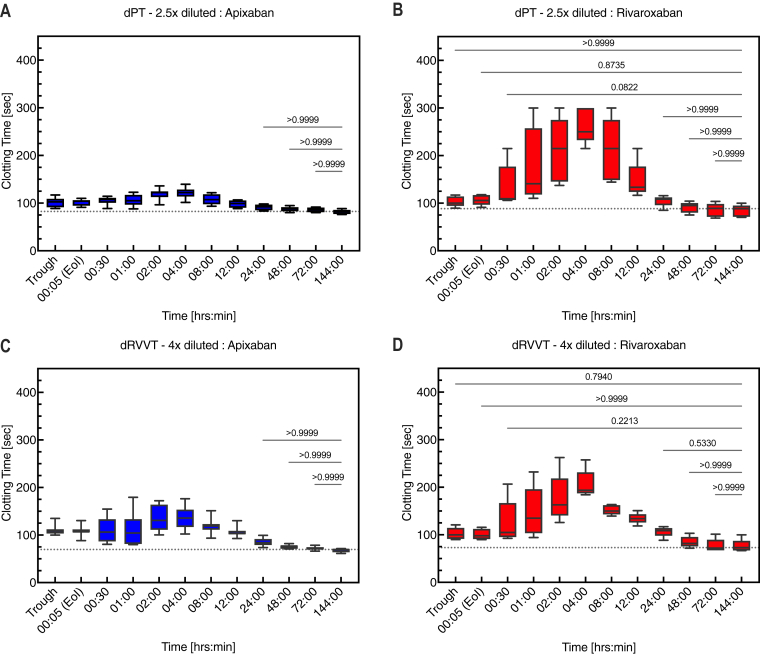
Time course of dPT assay results in healthy older subjects who received apixaban (A) or rivaroxaban (B) and time course of dRVVT assay results in subjects who received apixaban (C) or rivaroxaban (D). dPT and dRVVT were measured in 2.5× and 4× diluted plasma, respectively, in samples taken from healthy subjects who received apixaban (5 mg twice daily) or rivaroxaban (20 mg once daily) for 3.5 days, followed by a single intravenous infusion of placebo for 5 minutes, started 5 minutes after the last dose of FXa DOAC on day 4. Citrated platelet-poor plasma samples were obtained immediately before the final dose of apixaban or rivaroxaban (trough), at the end of placebo infusion (00:05 [EOI]), and at various times up to 144 hours after the start of infusion. The dotted line represents the median level at 144 hours when the effects of apixaban or rivaroxaban had worn off. The data at each time point were compared with those at 144 hours using the paired Friedman test; all comparisons were significant (*P* < .05, not indicated in the figure) except for those indicated in the figure. DOAC, direct oral anticoagulant; dPT, dilute prothrombin time; dRVVT, dilute Russell viper venom time; FXa, factor Xa.

**Figure 7:**
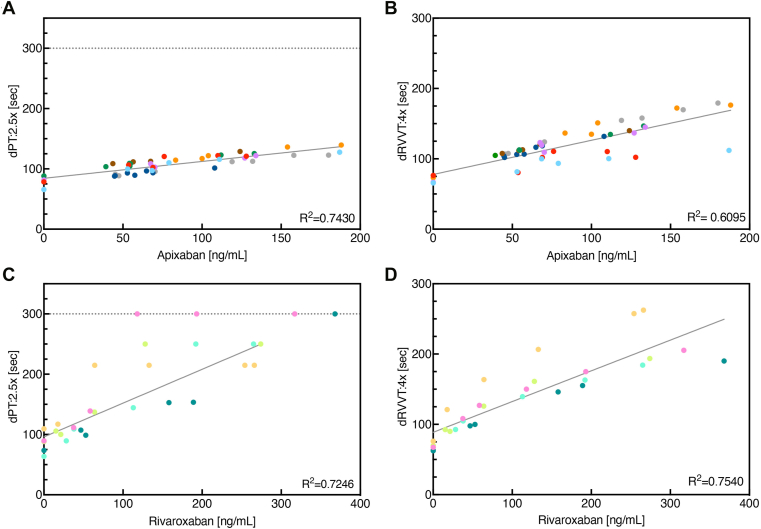
Correlation between dPT and dRVVT clotting times in healthy older subjects who received apixaban (top panels; *n* = 8 with 49 points) or rivaroxaban (bottom panels; *n* = 5 with 30 points) and apixaban or rivaroxaban plasma levels measured using LC-MS/MS. Individual subjects are represented by different colored points. dPT, dilute prothrombin time; dRVVT, dilute Russell viper venom time; LC-MS/MS, liquid chromatography-tandem mass spectrometry.

In healthy older subjects, the modified dPT and dRVVT assays performed after 3.5 days of treatment with apixaban or rivaroxaban showed that the clotting time was slightly prolonged before the final dose (trough) of apixaban or rivaroxaban relative to the clotting time at 144 hours, when the FXa DOAC would be expected to have been eliminated from the plasma (Figure 6). As expected, dPT and dRVVT clotting times increased over time after the final dose of apixaban or rivaroxaban and peaked at about 4 hours postdose. The peak in clotting time prolongation also corresponded with the peak concentration of FXa DOAC in plasma at 4 hours as measured by LC-MS/MS (data not shown). Finally, there was a positive correlation between clotting times (dPT or dRVVT) in plasma samples taken from healthy older subjects treated with apixaban or rivaroxaban and plasma levels of apixaban or rivaroxaban (Figure 7). For the dPT 2.5× dilution, *R*^2^ values were 0.7430 and 0.7246 for apixaban and rivaroxaban, respectively, and for the dRVVT 4× dilution, *R*^2^ values were 0.6905 for apixaban and 0.7540 for rivaroxaban.

**Figure 8:**
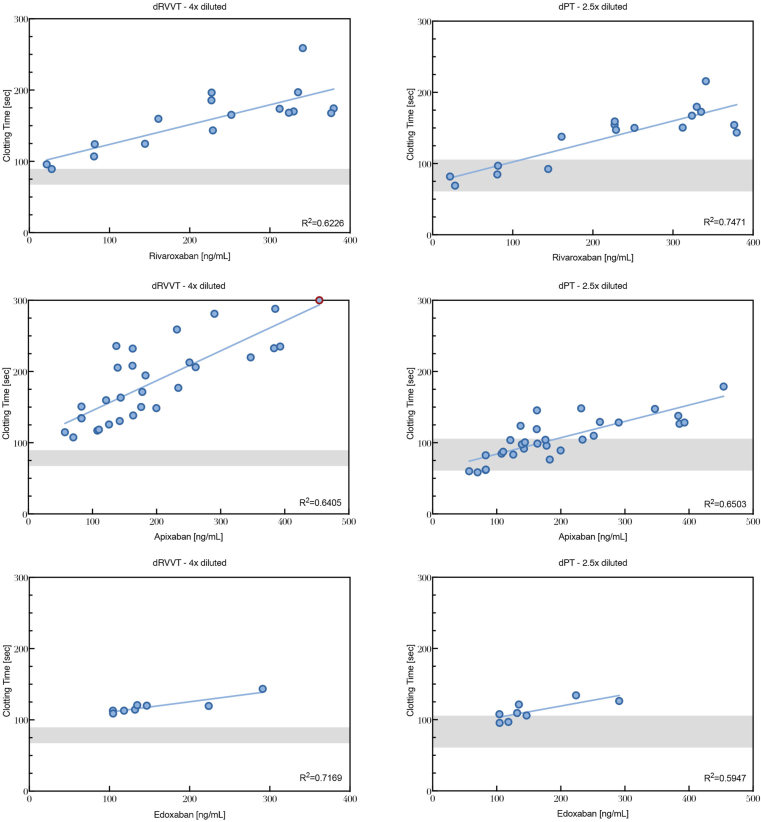
Correlation between dPT and dRVVT clotting times in nursing home residents, who were receiving rivaroxaban (top panels; *n* = 17), apixaban (middle panels; *n* = 30), or edoxaban (bottom panels; *n* = 8). The gray areas in each panel indicate the values obtained from healthy volunteers. Plasma concentrations of rivaroxaban, apixaban, and edoxaban were measured with the Ratachrom/STA-Liquid anti-Xa reagent on a Siemens CS-2500 coagulation analyzer. dPT, dilute prothrombin time; dRVVT, dilute Russell viper venom time.

In plasma samples from nursing home residents receiving FXa DOACs, the 4× diluted modified dRVVT assay was above the in-house applied normal range in all subjects receiving rivaroxaban (plasma concentration: 21-379 ng/mL, *n* = 9), edoxaban (plasma concentration: 104-291 ng/mL, *n* = 9), or apixaban (plasma concentration: 57-392 ng/mL, *n* = 30). Furthermore, clotting times showed good correlation with FXa DOAC levels for all 3 FXa DOACs (Figure 8). While the dPT assay represented good correlation with all 3 FXa DOACs, the presence of FXa DOACs, relative to the in-house normal range, was only confirmed at relatively higher levels of FXa DOAC (Figure 8).

## 4 Discussion

We modified 2 commercially available coagulation tests (ACTICLOT dPT and HemosIL dRVVT Confirm) to determine their usefulness as laboratory assays for measuring bypassing of the anticoagulant activity of FXa DOACs by VMX-C001 in clinical studies. Our findings showed the modified dPT and dRVVT assays were valid and highly sensitive for detecting the anticoagulant effects of FXa DOACs in spiked plasma samples from healthy older subjects who received FXa DOACs and in nursing home residents receiving FXa DOACs. The dRVVT assay was more sensitive than the dPT assay and could detect the effects of FXa DOAC at threshold concentrations (25-50 ng/mL) [24]. Plasma dilution improved the sensitivity of the assays; the 2.5× dilution (dPT) and 4× dilution (dRVVT) were the most sensitive tests. VMX-C001 completely reversed the prolonged dPT and dRVVT in plasma samples spiked *in vitro* with increasing concentrations of FXa DOACs, including apixaban. In comparison, andexanet fully reversed the prolonged clotting times with lower concentrations of FXa DOACs but dPT and dRVVT remained prolonged at higher concentrations of FXa DOACs. Full reversal across all FXa DOAC concentrations was only achieved with the highest concentration of andexanet (200 μg/mL) and partial reversal occurred with the lower concentrations andexanet (50 and 100 μg/mL), especially at higher concentrations of the FXa DOACs. These lower concentrations of andexanet represent clinically relevant levels [25]. In contrast, 4F-PCC only partially reversed the prolonged clotting times, irrespective of the type and dose of FXa DOAC or concentration of 4F-PCC used. Taken together, these results support the use of these modified dPT and dRVVT assays as valid and sensitive assays to monitor the bypassing of anticoagulation with VMX-C001 in clinical trials of patients with acute bleeding or of those who require urgent surgery during treatment with FXa DOACs. Finally, VMX-C001 restored dPT and dRVVT clotting times independent of FXa DOAC type or plasma concentration, highlighting its potential as general bypassing agent.

In urgent/emergency situations for patients on FXa DOACs, it is important to determine residual anticoagulant activity or FXa DOAC plasma levels to guide clinical decision making and avoid unnecessary administration of reversal agents [26]. Guidelines recommend that reversal agents should be administered if FXa DOAC plasma concentrations are > 30 ng/mL in patients requiring urgent interventions associated with a high risk of bleeding and > 50 ng/mL in patients with severe bleeding [17,24]. However, available tests for assessing FXa DOAC plasma concentrations have limitations [5,12,14,15,21,[26], [27], [28]]. Factor Xa DOACs interfere with almost all coagulation tests and their effect varies depending on the FXa DOAC used, its concentration, and the coagulation assay and reagent used [12,26]. In addition, chromogenic anti-FXa assays may not be suitable for use in emergency situations where the specific FXa DOAC used by a patient is unknown or the test results are not rapidly available [12]. Moreover, anti-FXa assays are not suitable for measuring residual anti-FXa activity after andexanet administration because the high sample dilution can give erroneously high anti-FXa levels [29]. The mechanism of action of VMX-C001, and the fact it is a bypassing agent and not a chelating agent, also mean that chromogenic anti-FXa assays are not suitable for use with VMX-C001. Point-of-care coagulation tests for FXa DOACs are of growing interest and may be a potential future solution for determining the need for a reversal agent [[30], [31], [32]].

Some researchers have suggested that dPT is a potentially sensitive test that might reflect physiological conditions [33]; however, the detection limit for apixaban was at concentrations of 42 ng/mL [21], and sensitivity was not improved by dilution of the thromboplastin reagents [14]. dRVVT assays have been reported to detect the effects of clinically relevant concentrations of FXa DOACs in patient plasma samples and good correlations have been reported between dRVVT and FXa DOAC plasma concentrations measured using anti-FXa assays [34,35] or LC-MS/MS [36]. However, an unmodified dRVVT test being investigated for use as a simple coagulation test for emergency situations, using *ex vivo* samples from patients treated with rivaroxaban or apixaban, was not sufficiently sensitive for reliable detection of clinically relevant FXa DOAC plasma concentrations [37].

Our *in vitro* assay validity experiments confirmed that the modified ACTICLOT dPT and HemosIL dRVVT Confirm assays had good reproducibility (CV <5% for dRVVT and <7% for dPT) and that prolonged clotting times (dPT and dRVVT) in the presence of apixaban were restored when VMX-C001 was present in the plasma sample. Our *in vitro* sensitivity experiments showed that FXa DOACs, including apixaban, prolonged dPT and dRVVT in a concentration-dependent manner and that the dRVVT assay was sensitive at a low drug level (< 50 ng/mL). Plasma dilution improved results from both assays. Although the dPT assay in undiluted and 2.5× diluted plasma spiked with apixaban (0-400 ng/mL) showed a dose-dependent increase in clotting times, it was not sensitive for detecting apixaban at threshold concentrations (< 50 ng/mL) as the dPT was within the control range of the assay (determined in the absence of apixaban) at low concentrations of apixaban.

When apixaban or rivaroxaban were orally administered to healthy older subjects, the dPT and dRVVT achieved peak prolongation at the same time (4 hours) as the peak FXa DOAC plasma concentration, which is consistent with their reported pharmacokinetic characteristics and time to maximum effect [5,6]. Our results showed a linear correlation between the prolongation of dPT or dRVVT in plasma samples taken from FXa DOAC–treated healthy older subjects and the plasma concentration of apixaban or rivaroxaban measured using LC-MS/MS. This confirms that both the dPT and dRVVT assays are sensitive for detecting FXa DOACs although there was more scatter in the data for rivaroxaban than apixaban. The data also showed variability in the clotting times (dPT and dRVVT) between individual subjects and suggested that some subjects may be more/less responsive to apixaban or rivaroxaban than others. We observed similar findings using plasma samples from nursing home residents on rivaroxaban, apixaban, and edoxaban. Further research is needed to explore the potential of these 2 assays to distinguish between high and low responders to FXa DOACs.

The differing effects of VMX-C001, andexanet, and 4F-PCC on FXa DOACs in these clot-based assays is expected based on their different mechanisms of action and biological effects. For example, andexanet is a modified, recombinant, inactive form of human FXa, which rapidly binds FXa inhibitor molecules and reduces anti-FXa activity [38]. It is approved in the United States and Europe for reversal of anticoagulation with apixaban or rivaroxaban in life-threatening/uncontrolled bleeding [39,40]. Andexanet is not approved for reversal of edoxaban (except in Japan) [41] or for the pretreatment of patients who require urgent surgery. However, data from 36 patients who received andexanet for reversal of edoxaban in the ANNEXA-4 study showed decreased anti-FXa activity and hemostatic efficacy similar to that obtained with apixaban and rivaroxaban [42]. Furthermore, current dosing recommendations of andexanet (low or high dose) limit its effective use as these are specific for each FXa DOAC and based on the dose and time since the last dose of apixaban/rivaroxaban, but this information is not always available [17].

PCCs are virally inactivated products containing concentrated amounts of vitamin K–dependent coagulation factors: 4F-PCC contains FII, FVII, FIX, and FX and is used off-label for the management of FXa DOAC–associated life-threatening bleeding. The mechanism by which 4F-PCC improves hemostasis in the presence of FXa DOACs is unclear. It has been suggested that the anticoagulant effects are overcome by the increasing levels of coagulation factors, leading to changes in thrombin generation [43], but this remains to be clinically proven [44]. 4F-PCC does not affect FXa DOAC concentrations [45] and has little effect on conventional clotting assays [46]. In contrast to andexanet, which immediately reverses all FXa DOAC–altered thrombin generation assay parameters, 4F-PCC inconsistently corrects/normalizes thrombin generation assay parameters independent of FXa DOAC concentrations [47]. Thus, 4F-PCC does not seem to function as an FXa reversal or bypassing agent [47].

Our results suggest that VMX-C001 can potentially be used as a workaround for LA testing when FXa DOACs are present. Currently, several methods are available for removing DOAC interference with coagulation assays, including DOAC-Stop and DOAC-Remove, but these all have limitations and may lead to incomplete DOAC removal, prolongation of clotting time or a procoagulant effect [48]. Furthermore, VMX-C001 may have a role for confirming the presence of FXa DOACs in plasma if this is required due to its ability to restore the dPT/dRVVT clotting time.

Strengths of our study include that dPT and dRVVT are methods where an actual clot is formed as readout. This has an advantage over chromogenic assays where only FXa activity is measured. dPT and dRVVT are rapid tests that require only basic coagulation equipment allowing for easy and cost-effective access to FXa DOAC testing for laboratories with limited resources. While the dRVVT assay starts with direct activation of FX, this test showed the highest sensitivity to the presence of FXa DOACs. On the contrary, the dPT assay also covers steps further up in the initiation phase of the coagulation pathway as it starts with TF-FVIIa, allowing for a more general assessment of coagulation. Another strength was the comparison of the effects of VMX-C001 with andexanet and 4F-PCC.

Potential limitations of our study include the low numbers of plasma samples for rivaroxaban and edoxaban. It is important to note that the sensitivity results may have been different if other reagents/kits were used. In addition, the impact of individual differences in specific coagulation factor activities on dPT and dRVVT readouts were not investigated. Such differences may, in part, explain the observed differences in patient response to FXa DOACs.

## 5 Conclusions

Our results have shown that the modified dRVVT and dPT assays can be used to assess reversal/bypassing of anticoagulation in patients treated with FXa DOACs, with a high degree of sensitivity. The clotting time results from healthy older subjects and nursing home residents receiving FXa DOACs correlated strongly with plasma drug concentrations. VMX-C001 bypassed all FXa DOACs and fully restored dRVVT and dPT clotting times from the prolonged times associated with the presence of FXa DOACs. Our findings suggest that the modified dPT and dRVVT assays can serve as surrogate end points in clinical studies of the FXa DOAC bypassing agent VMX-C001. They may also be used to monitor the effect of FXa DOAC anticoagulation and rescue agents such as andexanet and PCCs on coagulation.
