# Supplementary material for: Coagulation assays for assessing the bypassing of factor Xa direct oral anticoagulants using VMX-C001
Source: Res Pract Thromb Haemost. 2026 Feb 2;10(2):103375. doi: 10.1016/j.rpth.2026.103375 (PMC13053743; doi:10.1016/j.rpth.2026.103375)
Supplement: Supplementary Material [file mmc1.pdf]

Supplementary Material

Supplementary Table 1 Precision and accuracy of the modified dPT and dRVVT assay clotting times (sec) in NPP from healthy volunteers in the presence and absence of apixaban and/or VMX-C001

	Plasma dilution	Mean ± SD (sec)	Total CV (%)	Within-run CV (%)	Between-run CV (%)
**dPT assay**					
NPP	undiluted	44.2 ± 2.0	4.5	0.8	4.9
	2.5× diluted	85.5 ± 4.8	5.6	3.8	4.7
NPP + 100 ng/mL apixaban	undiluted	50.6 ± 2.5	4.9	0.9	5.4
	2.5× diluted	107.9 ± 5.7	5.3	2.7	5.2
NPP + 250 ng/mL apixaban	undiluted	59.3 ± 3.1	5.2	1.1	5.7
	2.5× diluted	137.8 ± 8.5	6.2	3.0	6.1
NPP + 250 ng/mL apixaban + 30 µg/mL VMX-C001	undiluted	40.7 ± 1.6	4.0	0.7	4.4
	2.5× diluted	74.7 ± 3.1	4.1	2.2	4.0
NPP + 30 µg/mL VMX-C001	undiluted	37.1 ± 1.5	4.0	0.8	4.3
	2.5× diluted	69.3 ± 2.8	4.1	2.3	3.8
**dRVVT assay**					
NPP	3× diluted	58.0 ± 1.4	2.3	1.4	2.2
	4× diluted	74.1 ± 3.0	4.0	3.1	3.1
NPP + 100 ng/mL apixaban	3× diluted	99.0 ± 3.7	3.8	1.7	3.8
	4× diluted	129.2 ± 5.3	4.1	2.5	3.7
NPP + 250 ng/mL apixaban	3× diluted	135.1 ± 5.3	3.9	1.6	4.0
	4× diluted	179.5 ± 7.8	4.3	2.7	3.9
NPP + 250 ng/mL apixaban + 30 µg/mL VMX-C001	3× diluted	66.8 ± 2.2	3.2	1.4	3.3
	4× diluted	80.6 ± 2.6	3.3	1.7	3.1
NPP + 30 µg/mL VMX-C001	3× diluted	60.2 ± 1.5	2.5	1.5	2.3
	4× diluted	73.1 ± 2.6	3.6	2.0	3.4

CV, coefficient of variation; dPT, dilute prothrombin time; dRVVT, dilute Russell's viper venom time; NPP, normal pooled platelet-poor plasma; SD, standard deviation.

Supplementary Figure 1 Dose-dependent prolongation of dPT in (A) undiluted and (B) 2.5× diluted plasma and of dRVVT in (C) 3× and (D) 4× diluted plasma with increasing concentrations of apixaban, edoxaban and rivaroxaban. Dotted lines represent maximal readout times for both assays.

dPT, dilute prothrombin time; dRVVT, dilute Russell's viper venom time.

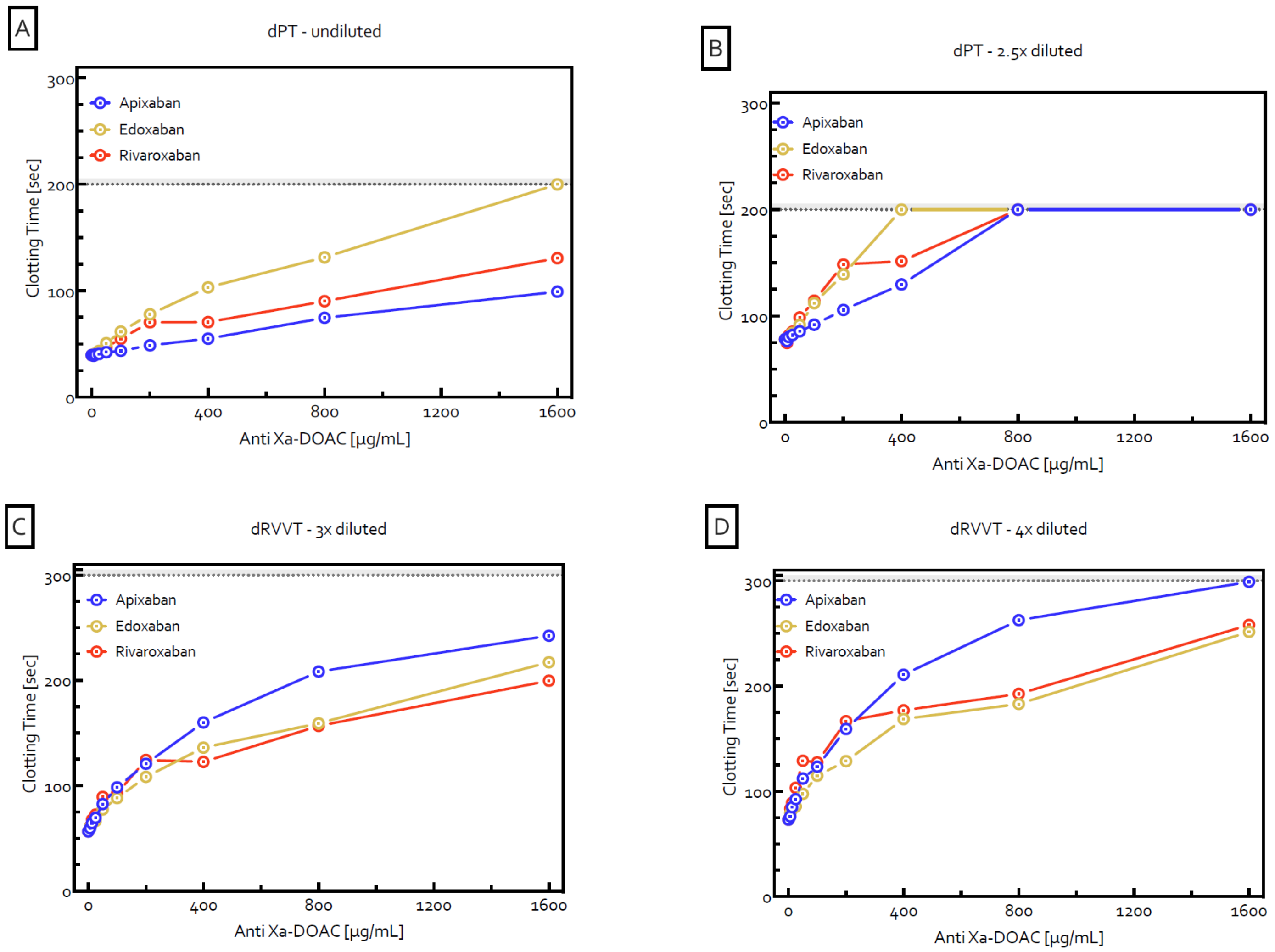

Supplementary Figure 2 Effect of FXa-DOACs on dPT and dRVVT assays in NPP. Clotting times were obtained in (A) undiluted and (B) 2.5× diluted plasma for dPT and in (C) 3× and (D) 4× diluted plasma for dRVVT in plasma spiked *in vitro* with rivaroxaban (n=3) across the dose range 0–400 ng/mL. Panels E–H show the clotting times in plasma spiked with edoxaban (n=3). The dotted lines represent the range of clotting times in the absence of the FXa-DOAC (control).

dPT, dilute prothrombin time; dRVVT, dilute Russell's viper venom time; FXa-DOACs, Factor Xa direct oral anticoagulants; NPP, normal pooled platelet-poor plasma.

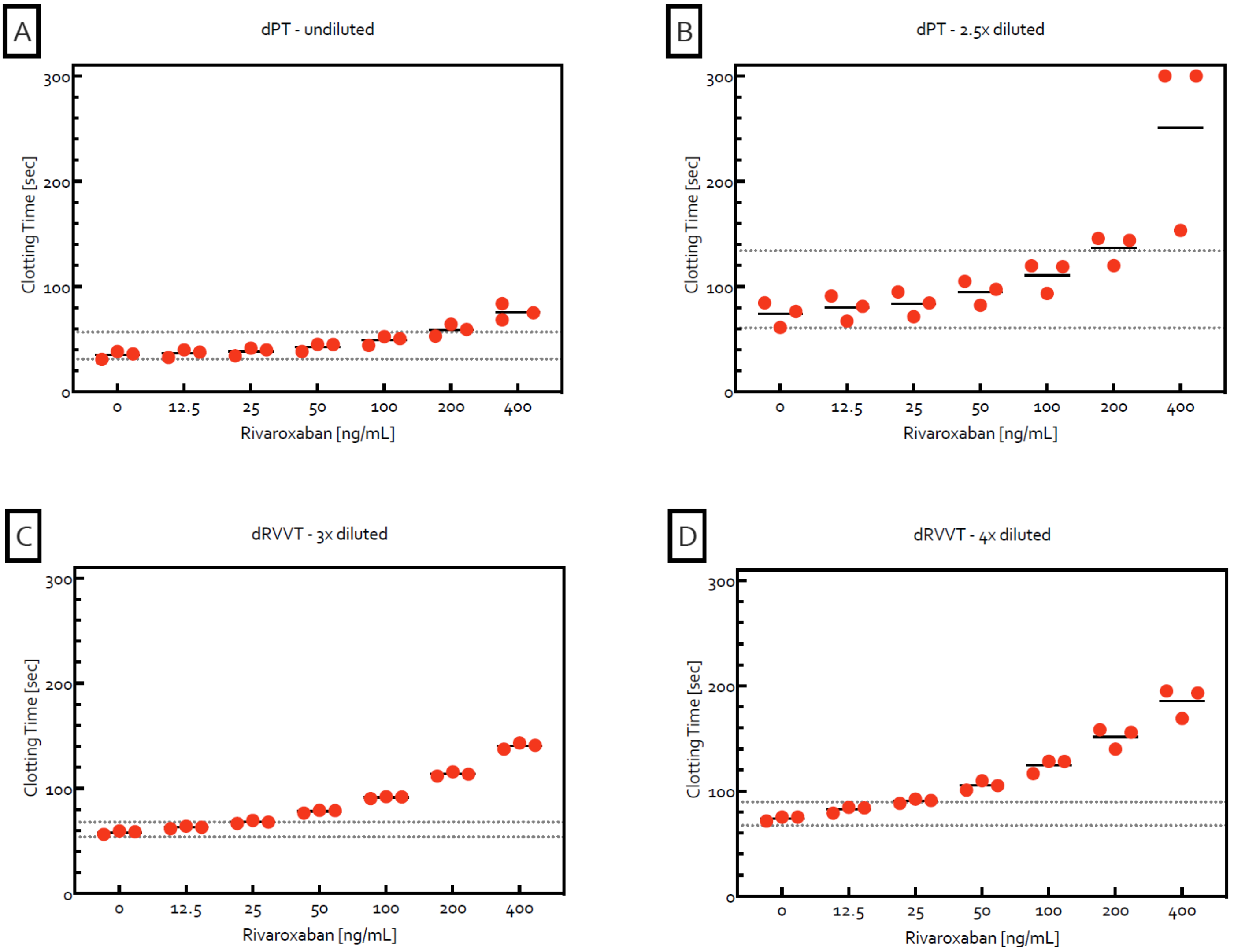

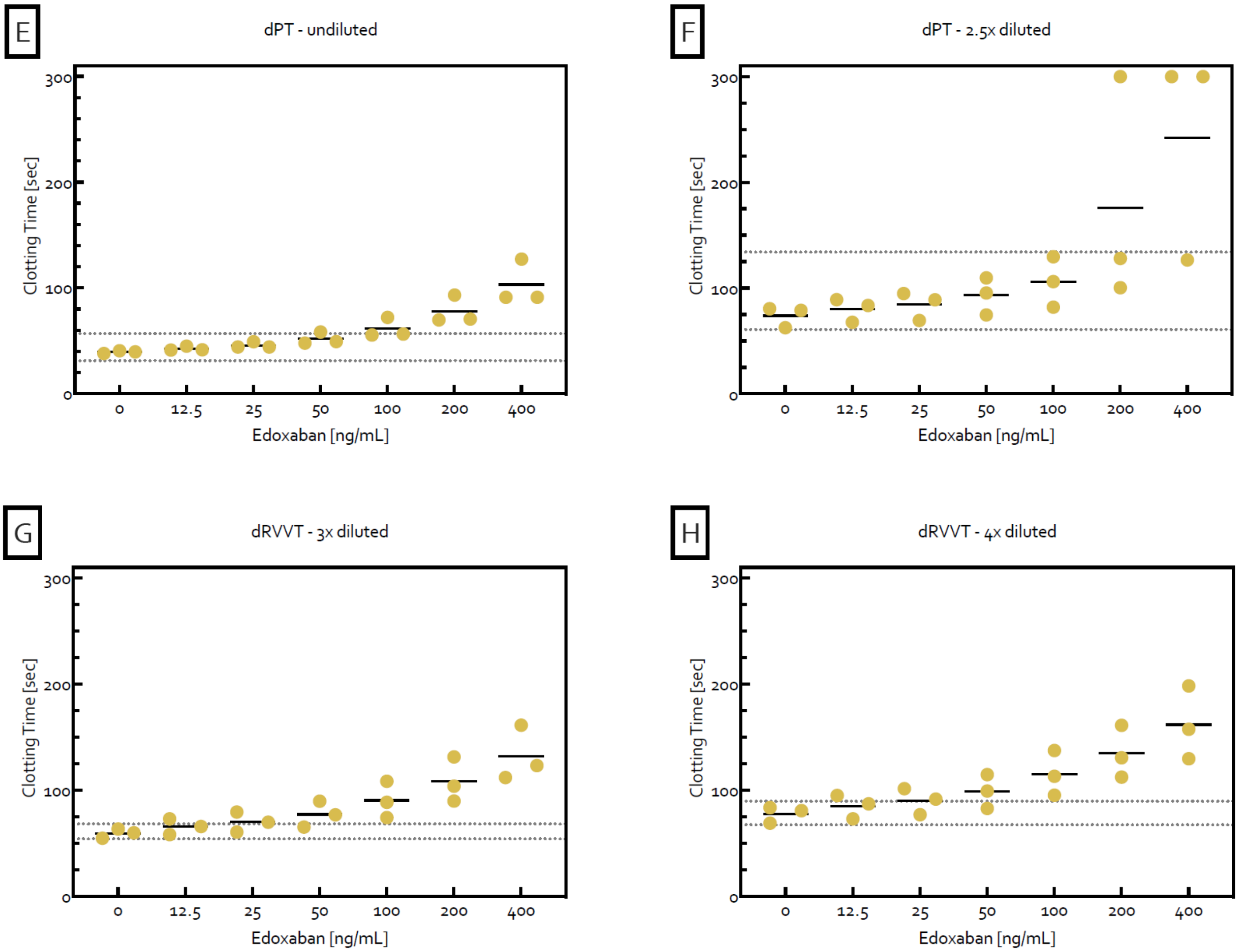
E
dPT - undiluted
F
dPT - 2.5x diluted
G
dRVVT - 3x diluted
H
dRVVT - 4x diluted
Clotting Time [sec]
0
100
200
300
0
12.5
25
50
100
200
400
Edoxaban [ng/mL]

Supplementary Figure 3 Validation of the dPT and dRVVT assays measured in 2.5× and 4× diluted plasma, respectively, in plasma samples spiked *in vitro* with rivaroxaban or edoxaban 0–400 ng/mL without (control) and with VMX-C001 (30 µg/mL). The clotting time upper limit was 300 sec. The dotted lines represent the min and max values for the control samples with 0 ng/mL FXa-DOAC.

dPT, dilute prothrombin time; dRVVT, dilute Russell's viper venom time; FXa-DOACs, Factor Xa direct oral anticoagulants.

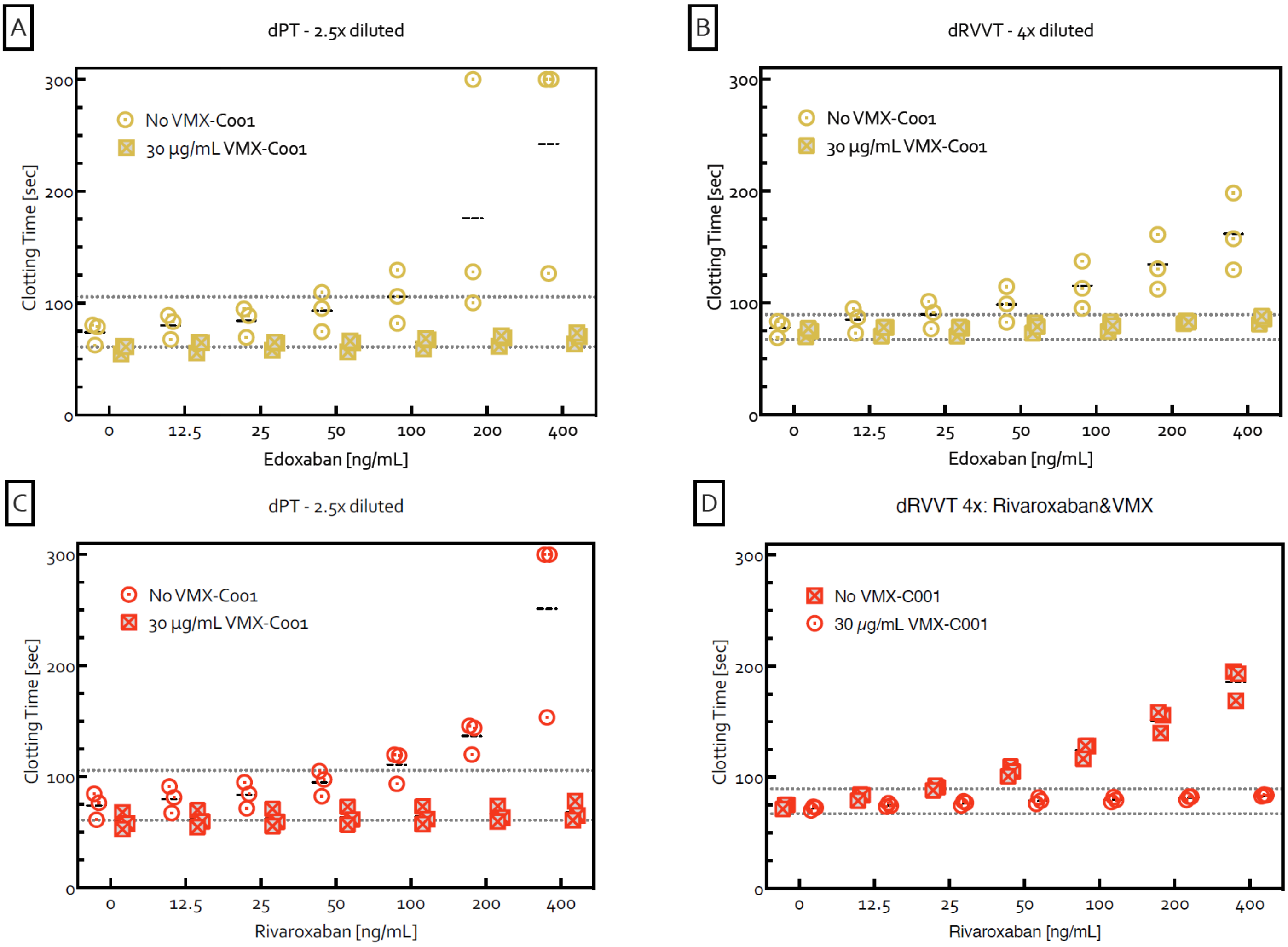

Supplementary Figure 4 Dose-dependent reversal by VMX-C001 (15, 30 and 60 µg/mL) and andexanet (50, 100 and 200 µg/mL) of the dose-dependent prolongation of dPT and dRVVT clotting times with edoxaban (0–1600 ng/mL). Dotted lines represent maximal readout times for both assays.

dPT, dilute prothrombin time; dRVVT, dilute Russell's viper venom time.

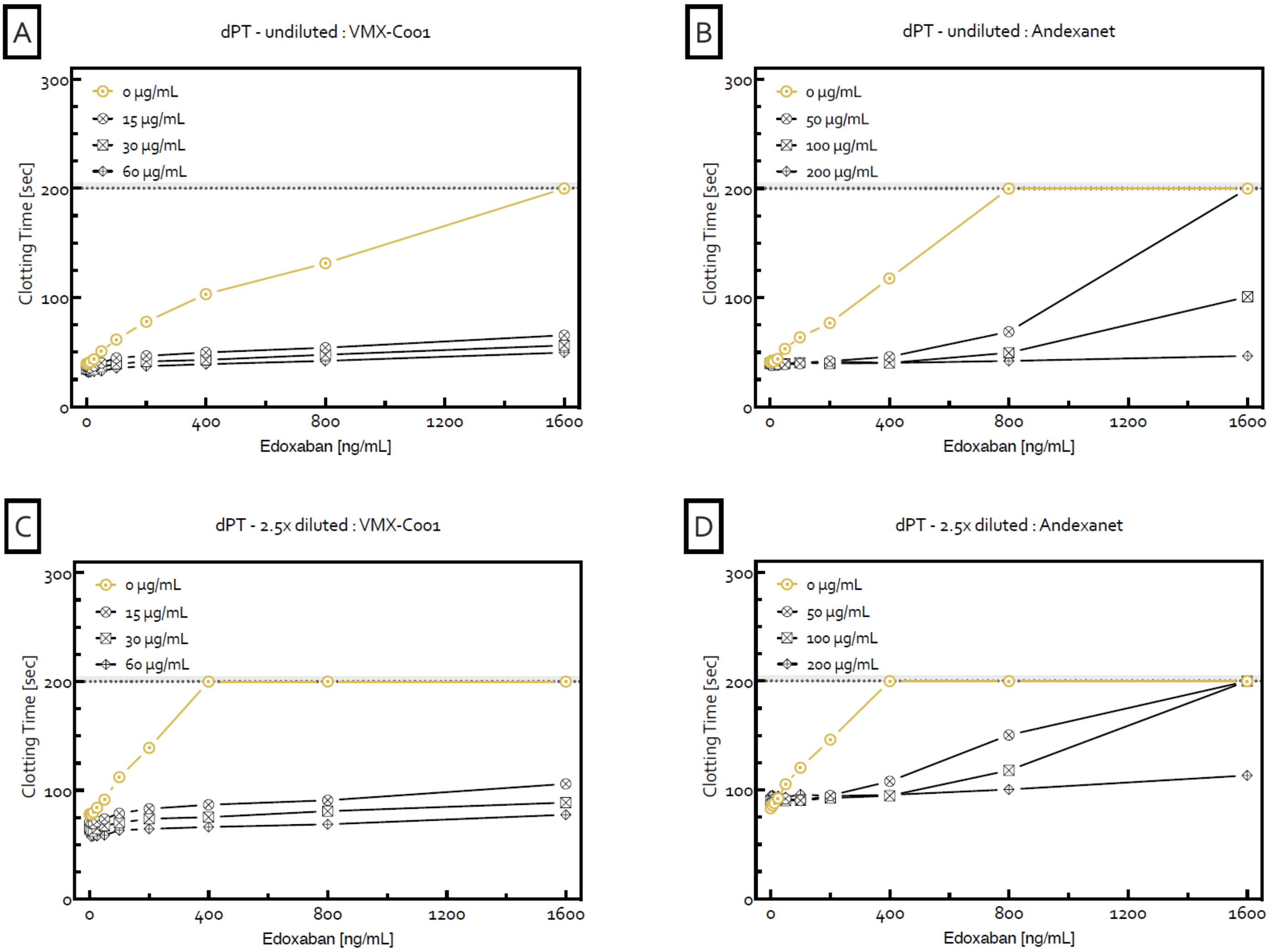

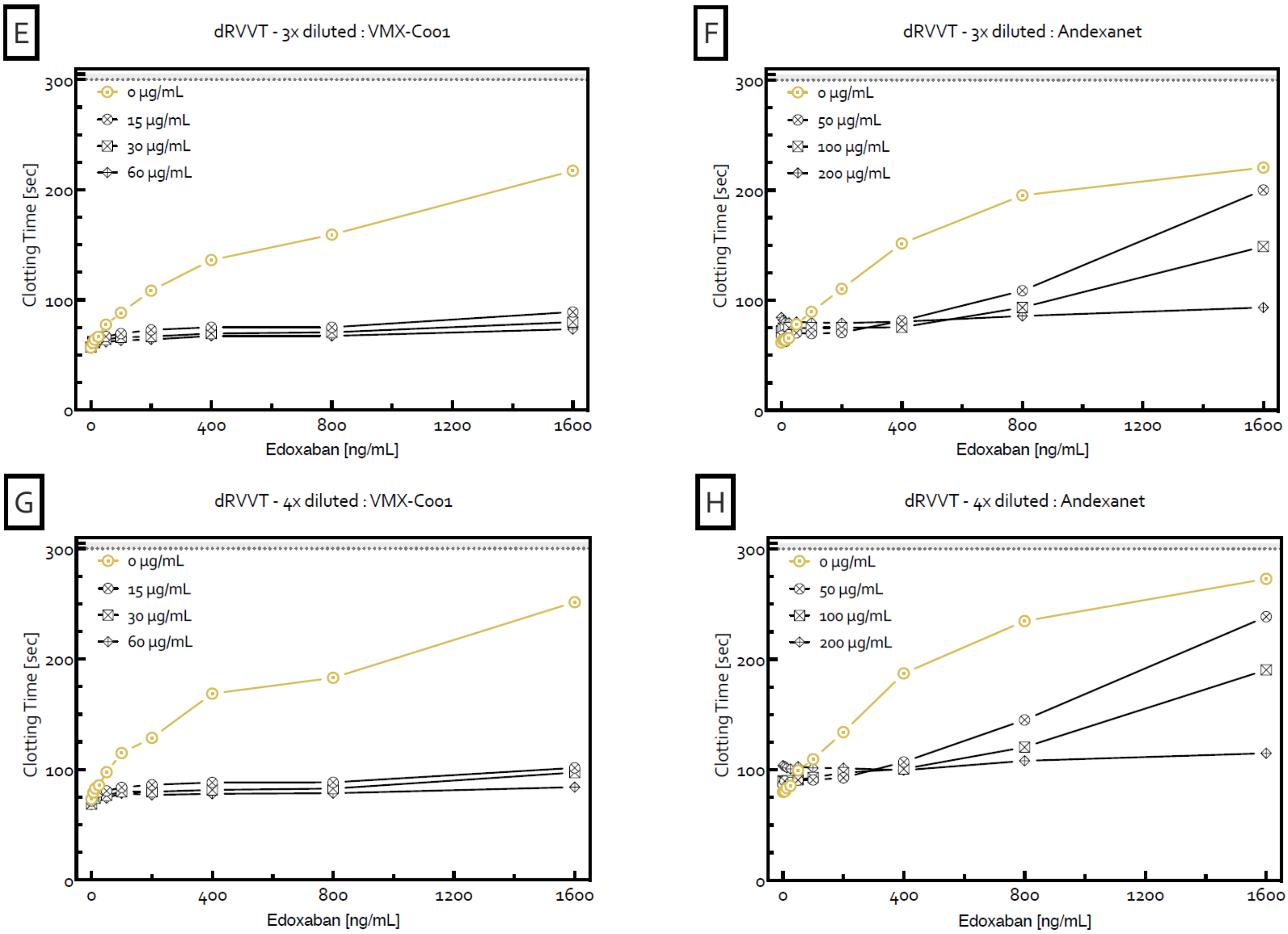
E
dRVVT - 3x diluted : VMX-C001
0 µg/mL
15 µg/mL
30 µg/mL
60 µg/mL
Clotting Time [sec]
Edoxaban [ng/mL]
F
dRVVT - 3x diluted : Andexanet
0 µg/mL
50 µg/mL
100 µg/mL
200 µg/mL
Clotting Time [sec]
Edoxaban [ng/mL]
G
dRVVT - 4x diluted : VMX-C001
0 µg/mL
15 µg/mL
30 µg/mL
60 µg/mL
Clotting Time [sec]
Edoxaban [ng/mL]
H
dRVVT - 4x diluted : Andexanet
0 µg/mL
50 µg/mL
100 µg/mL
200 µg/mL
Clotting Time [sec]
Edoxaban [ng/mL]

Supplementary Figure 5 Dose-dependent reversal by VMX-C001 (15, 30 and 60 μg/mL) and andexanet (50, 100 and 200 μg/mL) of the dose-dependent prolongation of dPT and dRVVT clotting times with rivaroxaban (0–1600 ng/mL). Dotted lines represent maximal readout times for both assays.

dPT, dilute prothrombin time; dRVVT, dilute Russell's viper venom time.

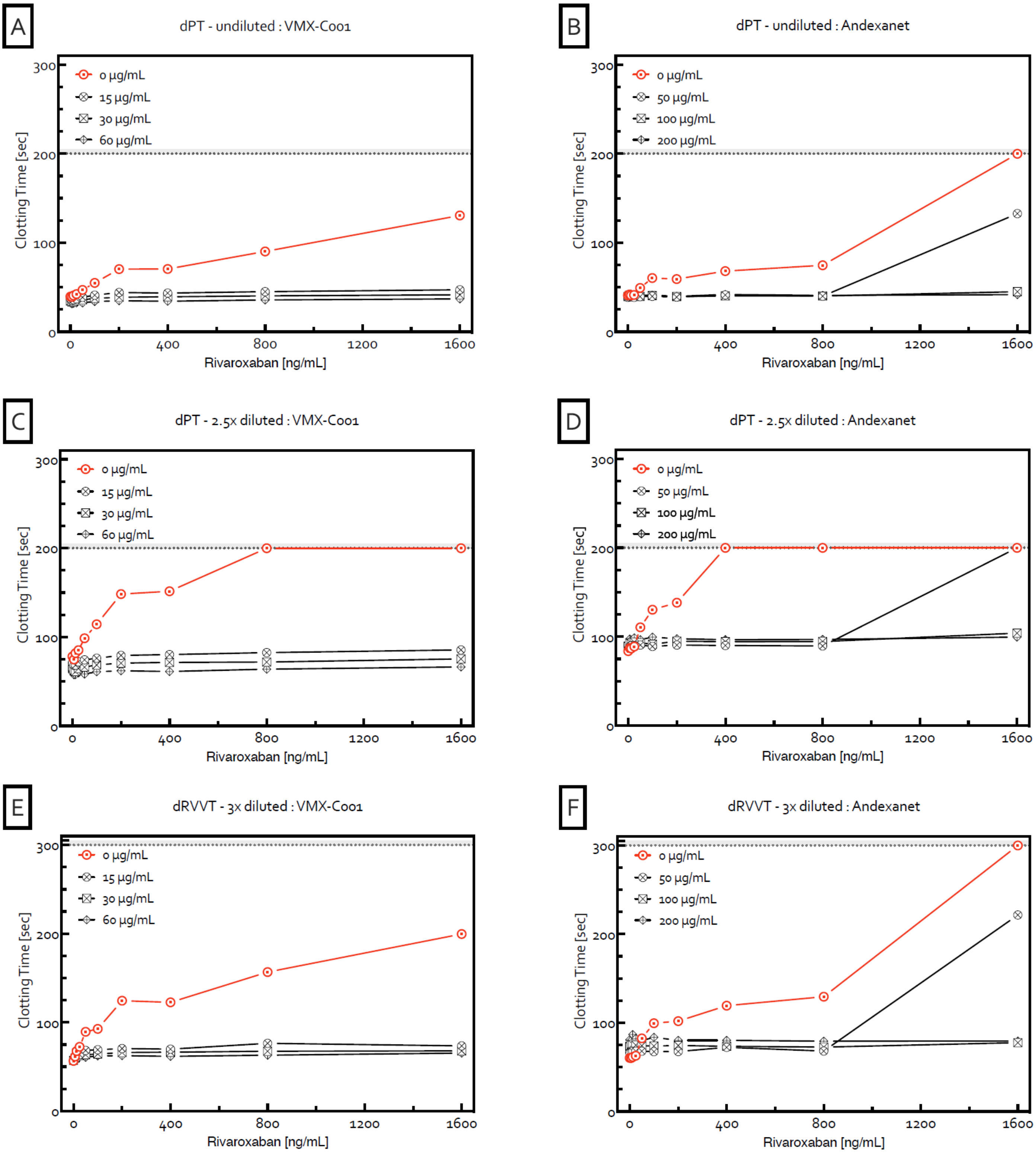

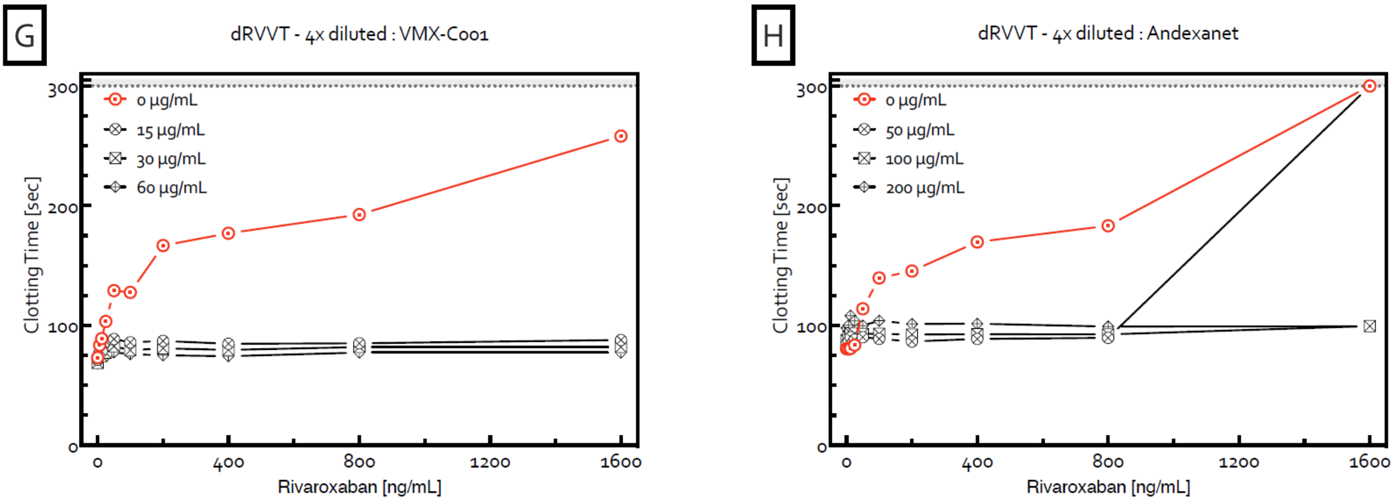
G
dRVVT - 4x diluted : VMX-C001
0 µg/mL
15 µg/mL
30 µg/mL
60 µg/mL
Clotting Time [sec]
Rivaroxaban [ng/mL]
H
dRVVT - 4x diluted : Andexanet
0 µg/mL
50 µg/mL
100 µg/mL
200 µg/mL
Clotting Time [sec]
Rivaroxaban [ng/mL]
